# The impact of overseas assignments on metabolic factors: Panasonic cohort study 23

**DOI:** 10.1093/joccuh/uiae071

**Published:** 2024-11-21

**Authors:** Tetsuya Murano, Hiroshi Okada, Masahide Hamaguchi, Kazushiro Kurogi, Yoshihide Tatsumi, Hiroaki Murata, Naoki Yoshida, Masato Ito, Michiaki Fukui

**Affiliations:** Department of Health Care Center, Panasonic Health Insurance Organization, 5-55 Sotojima-cho, Moriguchi 570-8540, Japan; Department of Endocrinology and Metabolism, Kyoto Prefectural University of Medicine, Graduate School of Medical Science, 465 Kajii-cho, Kawaramachi-Hirokoji, Kamigyo-ku, Kyoto 602-8566, Japan; Department of Endocrinology and Metabolism, Kyoto Prefectural University of Medicine, Graduate School of Medical Science, 465 Kajii-cho, Kawaramachi-Hirokoji, Kamigyo-ku, Kyoto 602-8566, Japan; Department of Health Care Center, Panasonic Health Insurance Organization, 5-55 Sotojima-cho, Moriguchi 570-8540, Japan; Department of Health Care Center, Panasonic Health Insurance Organization, 5-55 Sotojima-cho, Moriguchi 570-8540, Japan; Department of Orthopaedic Surgery, Matsushita Memorial Hospital, 5-55 Sotojima-cho, Moriguchi 570-8540, Japan; Department of Health Care Center, Panasonic Health Insurance Organization, 5-55 Sotojima-cho, Moriguchi 570-8540, Japan; Department of Health Care Center, Panasonic Health Insurance Organization, 5-55 Sotojima-cho, Moriguchi 570-8540, Japan; Department of Endocrinology and Metabolism, Kyoto Prefectural University of Medicine, Graduate School of Medical Science, 465 Kajii-cho, Kawaramachi-Hirokoji, Kamigyo-ku, Kyoto 602-8566, Japan

**Keywords:** Overseas assignment, Metabolic factor, Japanese

## Abstract

**Objectives:**

This study aimed to assess the effects of overseas assignments on the metabolic factors associated with lifestyle disease including body mass index, blood pressure, plasma glucose, lipid profiles, liver enzyme, and uric acid in Japanese individuals.

**Methods:**

A retrospective cohort study was conducted using annual health examination data from employees of the Panasonic Corporation in Japan. We evaluated the differences in the changes in metabolic factors associated with lifestyle disease during the observation periods between the overseas and non-overseas assignment groups. Propensity score matching was performed to match the characteristics of the two groups. In subgroup analysis, the impact of family accompaniment and the destination on metabolic factors associated with lifestyle disease were also evaluated.

**Results:**

The median ages of the overseas (n = 899) and non-overseas assignment groups (n = 899) were 46 (41–50) and 46 (41–50) years. The average overseas assignment duration was 4.1 ± 1.7 years. Overall, 65.4% of individuals were assigned overseas alone in the overseas assignment group. No significant differences were observed in the changes in metabolic factors associated with lifestyle disease between the overseas and non-overseas assignment groups. In subgroup analyses, the family accompaniment and the destination did not affect changes in any of metabolic factors associated with lifestyle disease during the overseas assignment.

**Conclusions:**

In conclusion, no significant difference was observed in metabolic factors associated with lifestyle disease between the overseas and non-overseas assignment groups in Japanese employees.

## Key points:

- **What is already known about this topic:** Japanese people migrate to Western countries experience an increase in obesity, deterioration of glucose tolerance, and progression of atherosclerosis.
- **What this study adds:** No significant change was observed in metabolic factors associated with lifestyle disease when comparing overseas assignments with an average of 4.1 years to non-overseas assignments in Japanese people.
- **How this study might affect research, practice, or policy:** The findings of this study can contribute to taking the necessary measures for the health management of overseas assignees.

## 1. Introduction

Recently, the number of Japanese people with lifestyle-related diseases has been increasing, and the shortening of healthy life expectancy and the resolution of medical expenses due to vascular complications associated with lifestyle-related diseases have become major challenges.^1^^,^^2^ The increase in lifestyle-related diseases, including diabetes, among Japanese people is thought to be influenced by the Westernization of lifestyle habits against a genetic background, where body mass index (BMI) and insulin secretion ability are lower than those in Westerners.^3^^,^^4^

Many Japanese companies are sending employees to different countries, and according to the Ministry of Foreign Affairs, the total number is said to exceed 700 000. Several studies have reported infectious disease incidence among overseas travelers.^5^^,^^6^ Additionally, when Japanese people migrate to Western countries, changes in dietary habits and a decrease in physical activity have been reported as factors leading to an increase in obesity, deterioration of glucose tolerance, and an increased risk of atherosclerosis.^7^^,^^8^ According to the Ministry of Foreign Affairs, it has been reported that 64.5% of the causes of death among Japanese expatriates are due to diseases, including cardiovascular disease.^9^ Therefore, it is important to consider the impact of overseas assignments on metabolic factors that can lead to cardiovascular disease among for Japanese companies. However, there are no studies examining the impact of a limited-term overseas assignment on weight and lifestyle-related diseases. By analyzing the data of overseas assignees and clarifying the impact on health, we believe we can contribute to taking the necessary measures for the health management of overseas assignees. We hypothesized that even within a limited period, overseas assignment would worsen metabolic factors associated with lifestyle disease including BMI, blood pressure, plasma glucose, lipid profiles, liver enzyme, and uric acid. Therefore, in this study, we used workplace data to examine the extent to which overseas assignments of Japanese people affect their metabolic factors associated with lifestyle disease.

**Figure 1:**
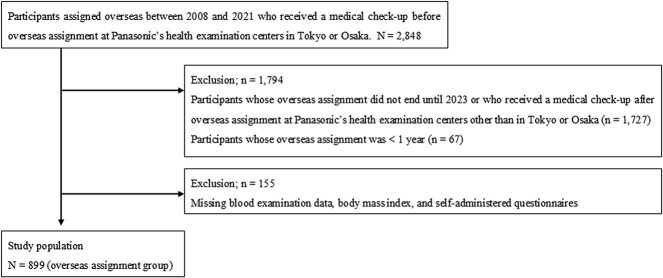
A flow diagram of the participant selection process.

## 2. Methods

### 2.1. Study design and ethical considerations

This retrospective cohort study was conducted using data from routine annual health examinations performed by Panasonic Corporation in compliance with the Industrial Safety and Health Act of Japan. The primary purpose of a health examination is to assess the health status of employees and identify risk factors for cardiovascular disease and lifestyle-related diseases. The employees involved in overseas assignments are required to undergo medical checkups before and after their assignments. Additionally, before overseas assignments, occupational health physician assessed their health based on the results of a health examination and, as needed, provided recommendations for medical consultations and guidance on improving lifestyle habits to the employees.

This study was approved by the Local Ethics Committee of the Panasonic Health Insurance Organization (approval number: 2024-001) and was conducted in accordance with relevant laws, institutional guidelines, and the principles of the Declaration of Helsinki. In accordance with the policy of the Ministry of Health, Labour and Welfare in Japan, informed consent was not obtained, and the study was an opt-out study.

### 2.2. Participants

This study included participants aged ≥18 years assigned overseas between 2008 and 2021, whose overseas assignment ended until 2023, whose overseas assignment was ≥1 year, and who received a medical check-up before and after overseas assignments at Panasonic’s health examination centers in Tokyo or Osaka. Participants with missing data on blood examinations, BMI, or self-administered questionnaires were excluded. A flow diagram of the participant selection process is shown in Fig. 1.

### 2.3. Observation period

The observation period was defined as the time from baseline to follow-up. The baseline was defined as the point at which they received the most recent examination before their overseas assignments. The follow-up was defined as the point at which the participants received the most recent examination after returning from overseas assignments.

### 2.4. Data collection

Data were collected from a health examination database between 2008 and 2023. The participants underwent height, weight, blood pressure, and waist circumference (WC) measurements, along with blood and urine sample examinations during health examinations before and after overseas assignments. Information regarding alcohol consumption and frequency; smoking status (present, past, or never); physical exercise habits; and history of the usage of antihypertensive, dyslipidemia, and antidiabetic medications was collected using a self-administered questionnaire in every health examination. Alcohol consumption was defined as an average daily alcohol intake ≥20 g, determined by the amount of alcohol consumed and the frequency of drinking. Regular exercisers were defined as participants who exercised regularly for a minimum of 30 min for ≥2 days/week for at least 1 year. Occupation types were defined as technical jobs, salespersons, manufacturers, office workers and others.

### 2.5. Definition of metabolic factors associated with lifestyle disease

We defined metabolic factors associated with lifestyle disease as BMI, WC, systolic blood pressure (SBP), diastolic blood pressure (DBP), aspartate aminotransferase (AST), alanine aminotransferase (ALT), fasting plasma glucose (FPG), triglycerides, high-density lipoprotein (HDL), low-density lipoprotein (LDL) and uric acid in our study.

**Table 1:** Characteristics of participants at baseline in overseas and non-overseas assignment groups.

	overseas assignment group	non-overseas assignment group	P value
N	899	899	
Male (%)	98.6%	98.6%	1.00
Age (y)	46 (41-50)	46 (41-50)	0.997
Body mass index (kg/m^2^)	23.4 (21.9-25.4)	23 (21.1-25)	0.03
Waist circumference (cm)	83.6 (78.2-88.9)	82 (76.9-88.7)	0.06
Systolic blood pressure (mmHg)	119 (110-127)	120 (111-128)	0.19
Diastolic blood pressure (mmHg)	75 (69-83)	75 (69-83)	0.81
Aspartate aminotransferase (U/L)	21 (18-26)	21 (18-26)	0.53
Alanine aminotransferase (U/L)	22 (16-31)	22 (16-31)	0.52
Fasting plasma glucose (mg/dL)	91 (86-97)	92 (87-98)	0.002
Triglycerides (mg/dL)	101 (71-153)	96 (70-143)	0.11
HDL cholesterol (mg/dL)	56 (48-65)	56 (47-66)	0.98
LDL cholesterol (mg/dL)	126 (106-146)	125 (106-144)	0.12
Uric acid (mg/dL)	6.3 (5.6-7.1)	6.2 (5.4-7.0)	0.004
Smoking(none/past/current), n,(%)	470/185/244 (52.3/20.6/27.1)	447/172/280 (49.7/19.1/31.1)	0.17
Alcohol consumption, n, (+)(%)	247 (27.5)	244 (27.1)	0.87
Physical exercise, n, (+)(%)	192 (21.4)	191 (21.2)	0.95
Occupation type, n, (%) Technical jobs/salesperson/ manufacturer/office worker/ other	371/139/51/246/92 (41.3/15.5/5.7/27.4/10.2)	372/139/51/246/91 (41.4/15.5/5.7/27.4/10.1)	1.00

Data are presented as median (interquartile range) or absolute number (percentage).

HDL, high density lipoprotein; LDL low density lipoprotein

### 2.6. Statistical analysis

Continuous variables are expressed as medians (interquartile ranges). Non-overseas assignees were selected from the Panasonic cohort study database, which conducts health examinations on approximately 140 000 individuals annually. In addition, to remove selection bias, propensity score matching was performed, and propensity score-matched cohorts (1:1 matching ratio) were built. Propensity scores were estimated controlling for sex, age, BMI, and occupation types. Matching was performed using the nearest-neighbor matching method with a caliper coefficient of 0.05. The observation period of the non-overseas assignment group was set to correspond to the duration of the overseas assignment of the assignees who were matched by propensity score matching.

The differences in baseline characteristics between the overseas and non-overseas assignment groups were evaluated using Student’s t-test, Mann–Whitney U test, or chi-square test. The comparison of metabolic factors associated with lifestyle disease at baseline and follow-up within the groups was performed using the Wilcoxon signed-rank test or paired t-test. Changes in metabolic factors associated with lifestyle disease factors from baseline to follow-up between the two groups were compared using the Mann–Whitney U test. If comparison was performed between the three groups, the analysis of variance or Kruskal-Wallis test was conducted. Given that lifestyle habits may change depending on the country of overseas assignment and whether family members accompany them, we conducted subgroup analyses. For the subgroup analysis, we assessed the differences in changes in metabolic factors associated with lifestyle disease from baseline to follow-up between a solo assignment and assignment with family in overseas assignment group. Additionally, we compared participants who were posted overseas to Europe or North America, those who were posted overseas to other regions, and non-overseas assignment group.

Differences were considered significant at p < 0.05. Statistical analyses were performed using JMP Pro software, version 17 (SAS Institute, Cary, NC, USA).

## 3. Results

The average duration of overseas assignments was 4.1 ± 1.7 years. The destinations for overseas assignments were North America (17.2%, n = 155), Central and South America (3.6%, n = 32), Europe (13.1%, n = 118), Asia (65.0%, n = 584), and other regions (1.1%, n = 10). Overall, 65.4% of the participants were assigned overseas alone. Table 1 shows the baseline characteristics of the overseas and non-overseas assignment groups. The median ages of the overseas and non-overseas assignment groups were 46 (41–50) and 46 (41–50) years, respectively. The baseline BMI and uric acid were higher in the overseas assignment groups than those in non-overseas assignment groups. The baseline glucose level was higher in the non-overseas assignment groups than those in overseas assignment groups.

Table 2 shows the comparison of BMI, WC, SBP, DBP, AST, ALT, FPG, TG, HDL cholesterol, LDL cholesterol, and uric acid at baseline and follow-up within the overseas and non-overseas assignment groups, and the change in those factors from baseline to follow-up between groups. We found significant differences in BMI, WC, SBP, DBP, AST, ALT, FPG, and HDL cholesterol at baseline and follow-up within both overseas and non-overseas assignment groups. However, no significant differences were observed in the changes in any of the factors between the two groups. Table 3 presents the subgroup analyses of the participants on a solo assignment or with family. Table 4 presents the subgroup analyses of the groups assigned to Europe or North America, and other regions. No significant differences were observed in changes in any of the factors from baseline to follow-up between the groups in any subgroup analysis.

**Table 2:** The comparison of metabolic factors associated with lifestyle disease in baseline and follow-up within overseas and non-overseas assignment groups and the change in those factors from baseline to follow-up between groups.

	overseas assignment group (n = 899)		non-overseas assignment group (n = 899)		P value
	baseline	follow-up point	baseline	follow-up point	
Body mass index (kg/m^2^)	23.4 (21.9-25.4)	23.8 (22-25.8)	23 (21.1-25)	23.2 (21.4-25.5)	
	<0.0001		<0.0001		0.66
Waist circumference (cm)	83.6 (78.2-88.9)	84.5 (78.9-90)	82 (76.9-88.7)	83.0 (77.5-89.8)	
	<0.0001		<0.0001		0.39
SBP (mmHg)	119 (110-127)	121 (113-128)	120 (111-128)	121 (113-129)	
	<0.0001		0.0001		0.77
DBP (mmHg)	75 (69-83)	78 (71-84)	75 (69-83)	78 (71-84)	
	<0.0001		<0.0001		0.51
AST (U/L)	21 (18-26)	22 (18-27)	21 (18-26)	22 (18-26)	
	<0.0001		0.007		0.35
ALT (U/L)	22 (16-31)	23 (16-35)	22 (16-31)	22 (16-32)	
	0.0009		0.04		0.35
FPG (mg/dL)	91 (86-97)	93 (88-99)	92 (87-98)	94 (88-100)	
	<0.0001		<0.0001		0.68
Triglycerides (mg/dL)	101 (71-153)	106 (72-153)	96 (70-143)	101 (69-152)	
	0.09		0.04		0.80
HDL cholesterol (mg/dL)	56 (48-65)	57 (49-66)	56 (47-66)	56 (47-67)	
	0.03		0.004		0.67
LDL cholesterol (mg/dL)	126 (106-146)	129 (107-149)	125 (106-144)	125 (105-145)	
	0.04		0.96		0.12
Uric acid (mg/dL)	6.3 (5.6-7.1)	6.3 (5.5-7.1)	6.2 (5.4-7.0)	6.1 (5.3-7.0)	
	0.10		0.15		0.90

Data are presented as median (interquartile range)

SBP, systolic blood pressure; DBP, diastolic blood pressure; AST, aspartate aminotransferase; ALT, alanine aminotransferase; FPG, fasting plasma glucose; HDL, high density lipoprotein; LDL low density lipoprotein

**Table 3:** The comparison of metabolic factors associated with lifestyle disease in solo overseas assignees and overseas assignment with family in overseas assignment group.

	solo overseas assignees (n = 588)		overseas assignment with family (n = 311)		P value
	baseline	follow-up point	baseline	follow-up point	
Body mass index (kg/m^2^)	23.6 (22-25.6)	23.8 (22.1-25.9)	23.5 (21.5-25.2)	23.8 (21.8-25.4)	
	<0.0001		0.0001		0.49
Waist circumference (cm)	84.2 (78.6-89.2)	84.8 (79.4-90.5)	83 (77-88)	83.5 (78.1-89.4)	
	<0.0001		0.0004		0.94
SBP (mmHg)	120 (111-128)	122 (113-129)	119 (110-125)	120 (112-128)	
	0.001		0.001		0.52
DBP (mmHg)	76 (70-83)	78 (72-84)	74 (68-82)	77 (71-84)	
	<0.0001		<0.0001		0.34
AST (U/L)	21 (18-26)	22 (18-27)	21 (19-25)	22 (19-27)	
	0.005		0.004		0.47
ALT (U/L)	22 (16-31.8)	23 (16-35)	22 (16-31)	23 (17-34)	
	0.006		0.03		0.81
FPG (mg/dL)	92 (86.3-98)	94 (88-100)	90 (85-95)	91 (87-97)	
	<0.0001		<0.0001		0.43
Triglycerides (mg/dL)	104.5 (73-162.8)	107 (73-158)	95 (63-133)	100 (69-144)	
	0.55		0.04		0.22
HDL cholesterol (mg/dL)	56 (48-66)	57 (48-66)	56 (48-64)	57 (49-65)	
	0.16		0.08		0.60
LDL cholesterol (mg/dL)	129 (108-147.8)	129 (109-151)	123 (103-144)	127 (104-145)	
	0.16		0.11		0.64
Uric acid (mg/dL)	6.3 (5.7-7.1)	6.3 (5.6-7.1)	6.2 (5.5-7.1)	6.3 (5.5-7.0)	
	0.08		0.68		0.48

Data are presented as median (interquartile range)

SBP, systolic blood pressure; DBP, diastolic blood pressure; AST, aspartate aminotransferase; ALT, alanine aminotransferase; FPG, fasting plasma glucose; HDL, high density lipoprotein; LDL low density lipoprotein

**Table 4:** The comparison of metabolic factors associated with lifestyle disease in participants who assigned overseas to Europe or North America, those to other regions and non-overseas assignment group.

	Europe or North America (n = 273)		Other regions (n = 626)		non-overseas assignment group (n = 899)		P value
	baseline	follow-up point	baseline	follow-up point	baseline	follow-up point	
Body mass index (kg/m^2^)	22.8 (21.2-25.2)	23.5 (21.7-25.3)	23.6 (22-25.5)	23.9 (22.1-26)	23.5 (21.5-25.2)	23.8 (21.8-25.4)	
	<0.0001		<0.0001		0.0001		0.39
Waist circumference (cm)	82 (76-88.5)	83.3 (78-89)	84.2 (79-89)	84.8 (79.5-90.5)	83 (77-88)	83.5 (78.1-89.4)	
	<0.0001		<0.0001		0.0004		0.46
SBP (mmHg)	117 (109-126)	120 (110-127.5)	120 (111-127)	122 (114-129)	119 (110-125)	120 (112-128)	
	0.007		0.0003		0.001		0.94
DBP (mmHg)	73 (66-82.5)	76 (70-83)	76 (70-83)	79 (72-84)	74 (68-82)	77 (71-84)	
	<0.0001		<0.0001		<0.0001		0.80
AST (U/L)	21 (18-26)	22 (19-27)	21 (18-25)	22 (18-27)	21 (19-25)	22 (19-27)	
	0.04		0.0008		0.004		0.65
ALT (U/L)	22 (16-33)	24 (16-34.5)	22 (17-31)	23 (16-35)	22 (16-31)	23 (17-34)	
	0.11		0.004		0.03		0.63
FPG (mg/dL)	90 (85-95)	92 (87-97)	92 (86-98)	93 (88-99)	90 (85-95)	91 (87-97)	
	<0.0001		<0.0001		<0.0001		0.87
Triglycerides (mg/dL)	95 (65.5-142)	100 (68.5-145.5)	105 (72.8-159)	106 (73-156)	95 (63-133)	100 (69-144)	
	0.006		0.76		0.04		0.13
HDL cholesterol (mg/dL)	57 (48-66)	57 (49-67)	56 (48-65)	57 (48.8-66)	56 (48-64)	57 (49-65)	
	0.20		0.08		0.08		0.90
LDL cholesterol (mg/dL)	125 (104-144)	129 (106-151.5)	127 (106.8-147)	129 (108-149)	123 (103-144)	127 (104-145)	
	0.16		0.12		0.11		0.28
Uric acid (mg/dL)	6.2 (5.5-7.1)	6.2 (5.3-7.1)	6.3 (5.7-7.1)	6.3 (5.6-7.1)	6.2 (5.5-7.1)	6.3 (5.5-7.0)	
	0.01		0.17		0.68		0.99

Data are presented as median (interquartile range)

SBP, systolic blood pressure; DBP, diastolic blood pressure; AST, aspartate aminotransferase; ALT, alanine aminotransferase; FPG, fasting plasma glucose; HDL, high density lipoprotein; LDL low density lipoprotein

## 4. Discussion

The major finding of this study was that there was no significant difference in the change from baseline to follow-up in BMI, waist circumference, SBP, DBP, AST, ALT, FBS, TG, HDL, LDL, and uric acid levels between the overseas and non-overseas assignment groups. In other words, overseas assignments did not affect these items, with an average duration of 4.1 years. Additionally, the presence or absence of family accompaniment and the destination did not affect changes in metabolic factors associated with lifestyle disease during the overseas assignment.

In previous reports targeting migrants to the United States, it was reported that when living in the United States for a long period, Japanese people who have migrated to the United States have a higher frequency of obesity,^10^ higher insulin resistance,^11^ lower adiponectin concentration,^11^ higher prevalence of metabolic syndrome and diabetes,^10^^,^^12^ and larger carotid intima-media wall thickness^13^^,^^14^ than Japanese people living in Japan, even though they are genetically the same. Changes in lifestyle habits are thought to influence overseas migration. Previous reports have shown that Japanese people who have migrated to the United States have a higher intake rate of animal protein, animal fat, and carbohydrates and less physical activity than Japanese people living in Japan. Differences in gut microbiota have also been suggested, with Japanese people who have migrated to the United States reporting a lower proportion of Bacteroidetes and a higher proportion of Firmicutes than Japanese people living in Japan^15^.

In this study, overseas assignments did not impact metabolic factors associated with lifestyle disease. Our results can be attributed to several reasons. It has been reported that there is a correlation between the duration of living abroad and obesity and diabetes risk in overseas migrants.^16^^,^^17^ However, the average duration of living abroad in this study was 4.1 years, which is short and may have been less likely to be affected by lifestyle habits. In addition, childhood and adolescent lifestyles affect dietary patterns^18^ and physical activity^19^ and have been reported to extend into adulthood. Therefore, lifestyle during childhood may be associated with obesity and other factors associated with metabolic syndrome in later years.^20-22^ Almost all the participants in this study spent their childhood in Japan; therefore, they may have been less likely to be influenced by dietary and exercise habits during their overseas assignments after becoming adults. In addition, in recent years, there have been Japanese restaurants abroad, and not only is Japanese food available there, but the homes of those assigned overseas are also equipped with cooking facilities. It is possible to cook Japanese cuisine based on what can be purchased in the market. Such an environment may have been a factor in not significantly changing their dietary habits when they were in Japan. Another possible factor is that the company conducted training and other programs related to dietary and exercise habits for those assigned overseas before their assignment. The fact that these assignees had received education may have influenced this study’s results.

In this study, we found significant differences in BMI, WC, SBP, DBP, AST, ALT, FPG, and HDL cholesterol at baseline and follow-up within both overseas and non-overseas assignment groups. Regardless of an overseas assignment, these factors have deteriorated over a period of 4.1 years. It has been reported that the weight of Japanese individuals increases over time in their 40s^23^, and that SBP rises by approximately 8 mmHg from their 40s to their 50s^24^. These age-related changes can explain the changes in metabolic factors associated with lifestyle disease observed over the 4.1-year period within both overseas and non-overseas assignment groups in this study.

This study had some limitations. Socioeconomic status might affect our results because the overseas assignments in company might be closely associated with socioeconomic status. Unfortunately, however, we have no data of socioeconomic status. Therefore, propensity scores were estimated controlling for occupation types which might be contributed to socioeconomic status. The duration between the health examination and the overseas assignment, and between returning to Japan and the next health examination might affect our result. Unfortunately, however, we have no these data. As we investigated only Japanese people, the results should be confirmed in other ethnic groups. Although no significant differences in all baseline characteristics except BMI, FPG and uric acid were found, therapeutic interventions might occur during overseas assignment. We have no data about therapeutic interventions during overseas assignment. Therefore, we could not eliminate this influence. Additionally, before overseas assignments, occupational health physician assessed employee health based on the results of a health examination and, as needed, provided recommendations for medical consultations and guidance on improving lifestyle habits to the employees. These interventions could influence our results. This was an observational study with a mean age of 4.1 years. If overseas assignments were extended over a longer period, there could be some impact on metabolic factors associated with lifestyle disease. Moreover, we excluded overseas assignment of less than one year because there is no comparable data of less than one year in non- overseas assignment group. These criteria might affect our results.

This study examined the impact of overseas assignment on metabolic factors associated with lifestyle disease. In conclusion, among Japanese people, no significant change was observed in metabolic factors associated with lifestyle disease when comparing overseas assignments with an average of 4.1 years to non-overseas assignments.

## Data Availability

The dataset of this study is available upon a reasonable request.
